# Synthetic Methods for the Preparation of Conformationally Restricted Analogues of Nicotine

**DOI:** 10.3390/molecules26247544

**Published:** 2021-12-13

**Authors:** Biswajit Panda, Gianluigi Albano

**Affiliations:** 1 Department of Chemistry, City College, 102/1 Raja Rammohan Sarani, Kolkata 700009, India; 2 Dipartimento di Chimica, Università degli Studi di Bari “Aldo Moro”, Via Edoardo Orabona 4, 70126 Bari, Italy

**Keywords:** catalytic processes, conformationally restricted analogues, nicotine, nitrogen heterocycles, synthetic methods

## Abstract

In the context of naturally occurring nitrogen heterocycles, nicotine is a chiral alkaloid present in tobacco plants, which can target and stimulate nicotinic acetylcholine receptors (nAChRs), a class of ligand-gated ion channels commonly located throughout the human brain. Due to its well-known toxicity for humans, there is considerable interest in the development of synthetic analogues; in particular, conformationally restricted analogues of nicotine have emerged as promising drug molecules for selective nAChR-targeting ligands. In the present mini-review, we will describe the synthesis of the conformationally restricted analogues of nicotine involving one or more catalytic processes. In particular, we will follow a systematic approach as a function of the heteroarene structure, considering: (a) 2,3-annulated tricyclic derivatives; (b) 3,4-annulated tricyclic derivatives; (c) tetracyclic derivatives; and (d) other polycyclic derivatives. For each of them we will also consider, when carried out, biological studies on their activity for specific nAChR subunits.

## 1. Introduction

**Figure 1:**
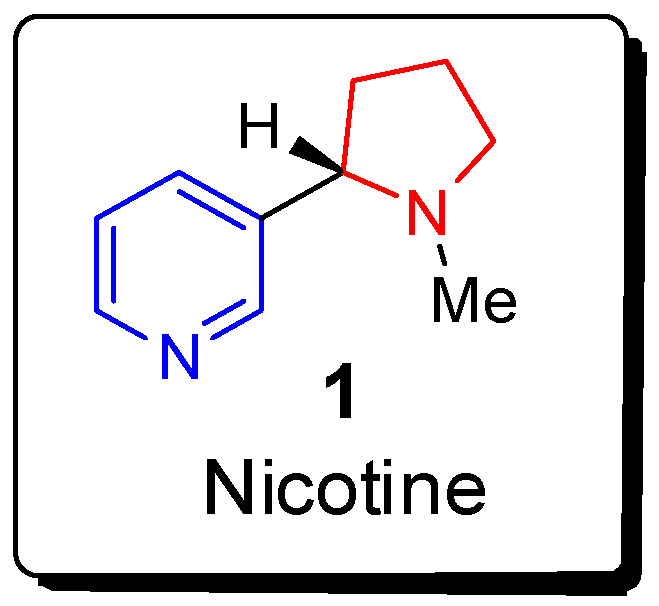
Chemical structure of nicotine (**1**), a chiral alkaloid consisting of two nitrogen heterocycles: a pyridine and a *N*-methylpyrrolidine moieties directly connected by a single carbon–carbon bond.

Nicotine (**1**) is a chiral alkaloid consisting of two nitrogen-containing heterocycles, i.e., a pyridine and a *N*-methylpyrrolidine moieties directly connected by a single C–C bond (Figure 1). In the context of naturally occurring *N*-heterocyclic compounds, nicotine is definitely one of the most known, because it is the most abundant alkaloid isolated from dried leaves of the tobacco plants *Nicotiana tabacum* and *Nicotiana rustica* [1,2]. Despite its potential pharmacological role in the treatment of Parkinson’s disease, Alzheimer’s disease, depression and other central nervous system-related disorders [3,4], clinical utility of nicotine is limited by its cardiovascular, gastrointestinal and neuromuscular side effects, and in particular by its high addiction liability [5,6].

**Figure 2:**
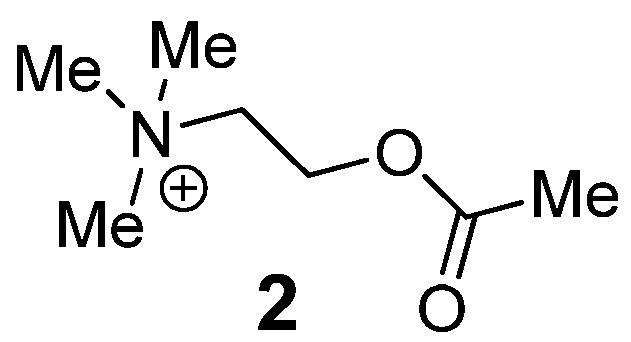
Chemical structure of the neurotransmitter acetylcholine (**2**).

The pharmacological features of nicotine (**1**) are closely related to nicotinic acetylcholine receptors (nAChRs), a class of ion channels present in both central and peripheral nervous systems that control synaptic activity [4]. The nAChRs are polypeptide receptors which typically respond to the neurotransmitter acetylcholine (**2**) (Figure 2), although they can also respond to drugs such as nicotine (**1**); in fact, there is considerable evidence that nicotine (**1**) can mimic the actions of acetylcholine (**2**) at the autonomic ganglia, the neuromuscular junction and some areas of the central nervous system [7]. Typically, nAChRs have a pentameric shape consisting of five subunits encoded by specific gene sequences; in particular, nine neuronal nAChR genes and three muscle nAChR genes have been identified to date, each of them indicated with a different Greek letter [8,9]. Neuronal nAChR genes are able to produce an enormous number of different pentameric combinations of nAChR receptors, located in many sites across the central and peripheral nervous systems, each of them with specific pharmacological roles; for example, the nAChR subunits α4β2 and α4β4 play a role in neurodegeneration and depression, the α3β2 subunit in dopamine release and Parkinson’s disease, the α3β4 subunit in norepinephrine release and in cardiovascular and gastrointestinal action.

**Figure 3:**
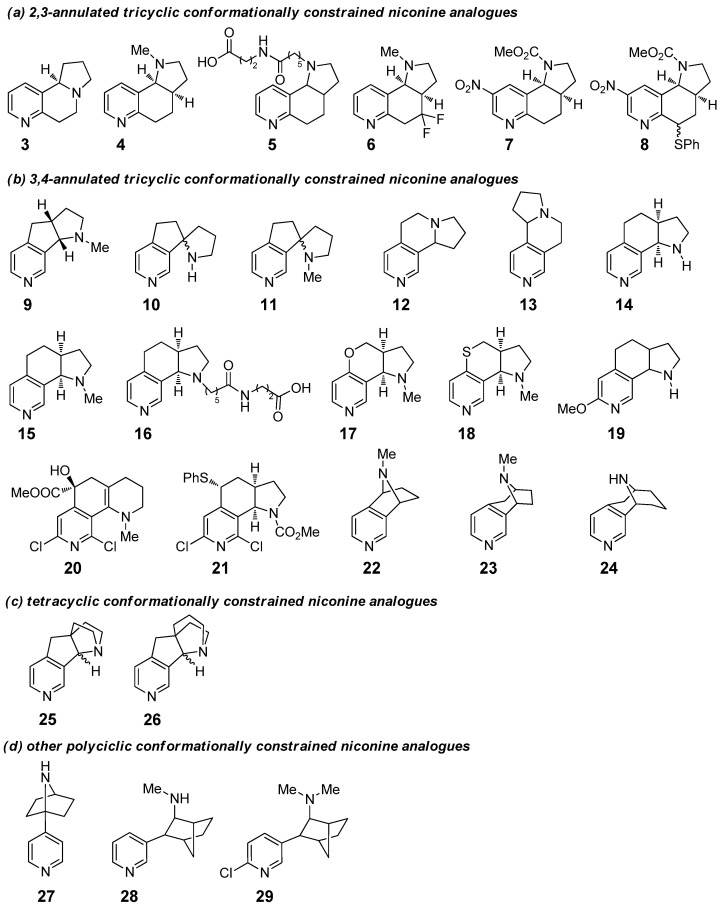
Conformationally restricted analogues of nicotine prepared by synthetic pathways involving one or more catalytic processes: (**a**) 2,3-annulated tricyclic derivatives (**3**–**8**); (**b**) 3,4-annulated tricyclic derivatives (**9**–**24**); (**c**) tetracyclic derivatives (**25**–**26**); and (**d**) other polycyclic structures (**27**–**29**).

Recently, several efforts have been made to the development of synthetic analogues of nicotine that are more selective for specific nAChR subunits [10]. In this context, a great interest is given by conformationally constrained derivatives, i.e., by altering the parent molecule in such a fashion that its original conformational mobility is severely limited to one particular conformation. The ability to “freeze out” the conformational dynamics of a ligand can improve its affinity and specificity for a receptor as a result of reducing the entropic loss upon binding. A variety of conformationally restricted analogues of nicotine have been successfully developed, by linking the pyridine and pyrrolidine moieties present in nicotine with a short chain, made of only carbon atoms or, in some cases, also bearing one or more heteroatoms. Some of them may also contain substituents on the pyrrolidine and pyridine ring, changes in the linker connecting the two heterocyclic rings, opening or replacement of the pyrrolidine ring, and in a few cases, the replacement of the pyridine scaffold with a suitable surrogate (Figure 3). All these conformationally constrained derivatives of nicotine have emerged among the most promising candidates for selective nAChRs-targeting ligands. In most cases, the preparation of these compounds involves one or more steps promoted by suitable catalytic systems: homogeneous catalysts, based on metal complexes with suitable ligands, as well as supported and recoverable catalysts.

Due to our recent interest in the development of efficient catalytic synthetic methods for the synthesis of heterocycles that have potential biologically active properties [11,12,13,14,15,16,17,18], in the present mini-review we will focus our attention only on the synthesis of those conformationally restricted analogues of nicotine involving one or more catalytic processes. In particular, below we will mostly follow a systematic approach as a function of the heteroarene structure, considering: (a) 2,3-annulated tricyclic derivatives; (b) 3,4-annulated tricyclic derivatives; (c) tetracyclic derivatives; and (d) other polycyclic derivatives (Figure 3). For each of these conformationally constrained nicotine derivatives we will describe the complete synthetic sequence(s) reported in literature, although we will try to give special focus to steps involving catalytic processes, or at least promoted by (sub)stoichiometric metal species used as additives; moreover, we will also briefly consider, when carried out, biological studies on their activity for specific nAChR subunits.

## 2. Synthetic Methods for the Preparation of Conformationally Restricted Analogues of Nicotine Involving Catalytic Processes

### 2.1. Synthesis of 2,3-Annulated Tricyclic Conformationally Constrained Nicotine Analogues

**Scheme 1:**
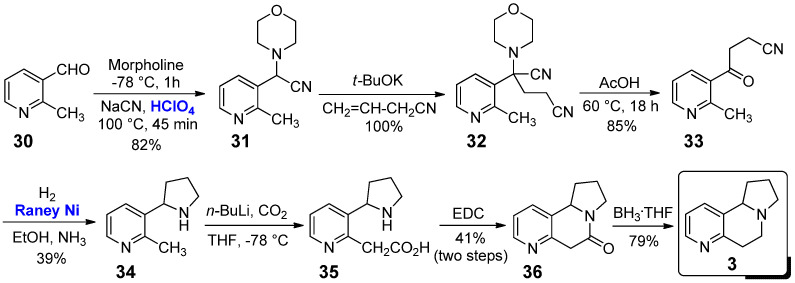
Synthesis of conformationally restricted nicotine analogue **3,** developed in 1978 by Catka and Leete [19]. The catalytic species involved in the synthetic pathway are indicated in blue.

The first class of conformationally constrained nicotine analogues, which we take into account in our mini-review, is that of 2,3-annulated tricyclic derivatives. To the best of our knowledge, the pyrido[2,3-*g*]indolizine **3** represents the first example of a synthetic “bridged” nicotine, whose preparation was reported in 1978 by Catka and Leete (Scheme 1) [19]. The authors started the synthesis from 2-methylpyridine-3-carboxaldehyde (**30**), which was converted into **31** using morpholine and NaCN, in the presence of catalytic amounts of HClO_4_; its use as a catalyst has been found to be fundamental in ensuring high yields of **31**. Its reaction with *t*-BuOK generated a stable anion, which was then treated with acrylonitrile to give the Michael addition product **32**. Acid mediated hydrolysis of **32** afforded the γ-ketonitrile **33**, followed by hydrogenation with H_2_ in the presence of Raney nickel, which was tested by the authors as the most powerful and easily removable (i.e., by a simple and fast filtration) catalytic system in allowing the formation of 2-methylnornicotine (**34**) in good yield. Subsequent lithiation of **34** and treatment with CO_2_ afforded the carboxylic acid **35**, which was then converted into tricyclic lactam **36** using EDC as activator. The final conformationally restricted nicotine analogue **3** was achieved in good yield (79%) by the reduction of lactam **36** using BH_3_·THF as the reducing agent. 

It is worth emphasizing that Catka and Leete were only interested in the synthesis of this nicotine analogue. In a following work by Martin and co-workers, the same compound **3** was synthesized as racemate following the same synthetic pathways, and then resolved into its enantiomers for a pharmacological comparisons to (+)- and (−)-nicotine (**1**) [20]. Unfortunately, they found that the biologic activity in several animal tissues of (+)-**3** and (−)-**3** was typically lower than the corresponding enantiomers of nicotine (**1**): in particular, in the contractions of the guinea-pig ileum, (−)-**3** (EC_50_ = 53 μm) was about 10-fold less potent than (−)-**1** (EC_50_ = 4.2 μm), while (+)-**3** (EC_50_ = 400 μm) was about 2-fold less potent than (−)-**1** (EC_50_ = 170 μm). These results clearly suggested that there may exist subpopulations of nicotine receptors to which both enantiomers of derivative **3** were not able to interact. In 2015, Kristensen et al. reported a study about the biological activity of the conformationally constrained nicotine derivative (*R*)-**12** (see Section 2.2 below), where racemic **3** was used as a negative comparison in the investigation of antagonist activity of the α4β2-subtype of nAChRs in whole cell of *Xenopus laevis* oocytes [21].

**Scheme 2:**
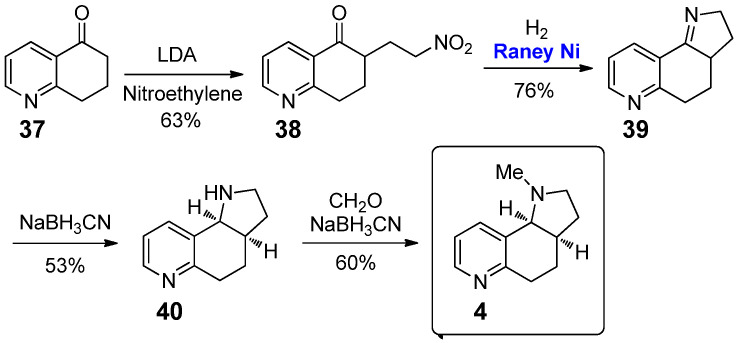
Synthesis of conformationally restricted nicotine analogue **4** developed in 1983 by Seeman and co-workers [22]. The catalytic species involved in the synthetic pathway are indicated in blue.

More extended studies have been performed on the pyrrolo[2,3-*f*]quinoline **4**. The original route to this 2,3-annulated tricyclic conformationally constrained nicotine analogue was reported in 1983 by Seeman and co-workers (Scheme 2) [22]. The synthesis began from the LDA mediated Michael-type addition of 7,8- dihydroquinolin-5(6*H*)-one **37** to nitroethylene. The following reduction of the nitro group of **38** was carried out by catalytic hydrogenation; Raney nickel (0.35 mol%) was here an efficient and clean catalyst, removed from the reaction mixture by filtration on Celite. The resulting amine moiety gave one-pot intramolecular condensation with the carbonyl group, affording imine **39** in 76% yield. Reduction of **39** with NaBH_3_CN afforded the corresponding amine **40** in 53% yield; finally, its methylation gave the nicotine derivative **4** in 60% yield. This route to pyrrolo[2,3-*f*]quinoline **4** has been followed for decades, as reported in 2002 by Crooks et al. [23] and in 2004 by Janda et al. [24]. However, in 2011 an alternative synthetic path for the preparation of **4**, starting from 2-methylpyridine, has been proposed by Crooks and co-workers, which is beyond the scope of our mini-review because it did not involve any catalytic step [25].

Several studies have been performed on the nicotine derivative **4** in order to evaluate its biological activity. In 2001, Crooks et al. reported for this compound a high binding affinity towards the α7 (K_i_ > 100 μM, assayed by inhibition of [^3^H]-MLA binding to rat brain membranes) and a moderate binding affinity towards the α4β2 (K_i_ = 12.2 μM, assayed by inhibition of [^3^H]-NIC binding to rat striatal membranes) nAChRs, which further increased by the complexation of the nitrogen atom of the pyridine scaffold with boron species (i.e., K_i_ > 100 μM for both α7 and α4β2 nAChRs) [26]. Further studies showed that *N*-quaternization of **4** could easily eliminate inhibitory activity at both α4β2* and α7* nAChRs, affording high affinity and selectivity for the nAChRs mediating nicotine-evoked dopamine release from striatum (IC_50_ values 30–310 nM) [27]. However, on the other hand the conformationally restricted nicotine analogue **4** did not show any agonist activity at α7 nAChRs receptors of rat expressed in *Xenopus* oocytes [28].

**Scheme 3:**
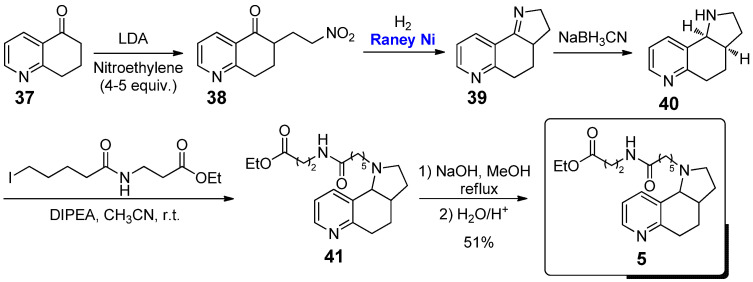
Synthesis of conformationally restricted nicotine analogue **5** developed in 2003 by Janda et al. [29]. The catalytic species involved in the synthetic pathway are indicated in blue.

The pyrrolo[2,3-*f*]quinoline **5** differs from **4** only by the different chain attached to the nitrogen atom of the pyrrolidine moiety. The synthesis, proposed in 2003 by Janda et al., was actually based on slight modifications of the above mentioned procedure reported by Seeman and co-workers for the preparation of **4** (Scheme 3) [29]. After obtaining the amine compound **40**, it was treated in acetonitrile and in the presence of diisopropylethylamine (DIPEA) with the suitable iodide of the desired linker to give ester **41**, which was then hydrolyzed to acid by saponification with NaOH in refluxing methanol to give the final nicotine analogue **5**.

From a biological point of view, Janda et al. used this compound as a constrained nicotine hapten, which was coupled to the carrier protein KLH, in order to study its immunogenic effects in light of a possible development of nicotine vaccines; interestingly, immunization of mice using a serum based on **5** resulted in high antibody titers (~25,000) and affinity for (*S*)-nicotine (K_d_ 0.60 ± 0.10 µM), nearly 2- and 3-fold improved with respect to their previous generation of serum based on other conformationally free nicotine analogues. The high biological interest of this nicotine derivative was further confirmed by its involvement in some patents focused on the development of nicotine vaccines [30,31].

**Scheme 4:**
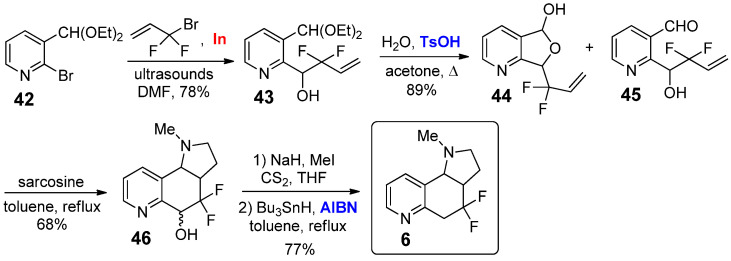
Synthesis of conformationally restricted nicotine analogue **6** developed in 2009 by Zhai et al. [32]. The catalytic species involved in the synthetic pathway are indicated in blue, and the stoichiometric metal species used as additives are depicted in red.

Another compound belonging to 2,3-annulated tricyclic conformationally constrained nicotine derivatives is the difluorinated pyrrolo[2,3-*f*]quinoline **6**, whose synthesis has been reported in 2009 by Zhai et al. (Scheme 4) [32]. The starting point was 2-bromopyridine **42**, subjected to an indium-promoted Barbier allylation with 3-bromo-3,3-difluoropropene in order to introduce a *gem*-difluoromethylene group; despite the presence of ultrasounds sonication, the use of indium as stoichiometric additive (1.2 equiv.) was found to be fundamental in order to obtain a high yield (78%) of **43**. The subsequent TsOH-catalyzed hydrolysis of diethyl acetal **43** afforded aldehyde **45**, which under thermal heating could partially evolve to the cyclic hemiacetal **44**. A sarcosine-promoted intramolecular azomethine ylide-alkene [3+2] cycloaddition was used to generate the tricyclic skeleton **46**, which was then subjected to the final step of dehydroxylation to give pyrrolo[2,3-*f*]quinoline **6**. It was performed through the formation of a xanthate (using NaH, methyl iodide and CS_2_ in THF), followed by the Barton–McCombie deoxygenation with Bu_3_SnH and catalytic amounts (20 mol%) of AIBN as a radical initiator, giving **6** in 77% yield. Despite the structural complexity of **6**, the use of many catalytic processes has allowed for the development of an appealing, few-step protocol for its preparation. Unfortunately, no studies of biological activity have been performed to date on such nicotine derivative.

**Scheme 5:**
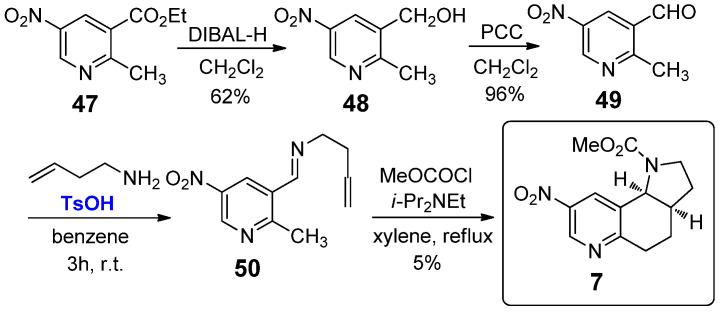
Synthesis of conformationally restricted nicotine analogue **7** reported in 2005 by Panda et al. [33]. The catalytic species involved in the synthetic pathway are indicated in blue.

Moving on the 2,3-annulated tricyclic conformationally constrained analogues bearing substituents on the pyridine scaffold, to the best of our knowledge only two compounds have been reported to date: the 8-nitro-1*H*-pyrrolo[2,3-*f*]quinolines **7** and **8**, synthesized in 2005 by Panda et al. starting from 2-methyl-5-nitronicotinate **47** [33]. Concerning the preparation of the nicotine derivative **7** (Scheme 5), the ester moiety of **47** was first reduced to the primary alcohol **48** by treatment with DIBAL-H at −78 ^°^C and then oxidized to aldehyde **49** by subsequent oxidation with PCC. Aldehyde **49** was converted into the corresponding imine **50** by the reaction with homoallyl amine in the presence of catalytic amounts (0.1 mol%) of TsOH. The final step involved the use of methyl chloroformate with DIPEA in refluxing xylene: a formal imine tautomerisation and intramolecular trapping allowed for the generation of a pyridyl *ortho*-quinodimethide, which evolved via intramolecular Diels–Alder reaction to nicotine analogue **7** in low yield.

**Scheme 6:**
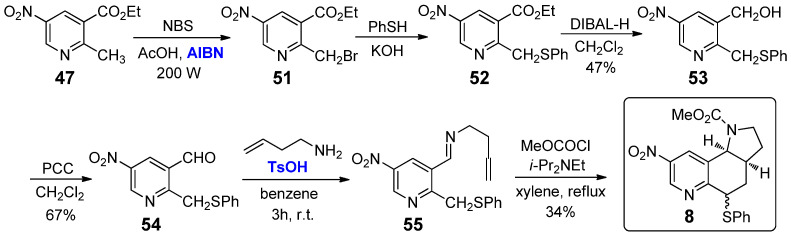
Synthesis of conformationally restricted nicotine analogue **8** reported in 2005 by Panda et al. [33]. The catalytic species involved in the synthetic pathway are indicated in blue.

The preparation of the 8-nitro-1*H*-pyrrolo[2,3-*f*]quinoline **8** was quite similar, although it consisted of two more initial steps (Scheme 6): (a) the benzylic bromination of 2-methyl-5- nitronicotinate **47** with NBS in the presence of catalytic amounts (0.5 mol%) of AIBN; (b) the nucleophilic substitution of **51** with thiophenol under basic conditions to give thioether **52**.

Although the presence of various functional groups in the chemical structure of the pyrrolo[2,3-*f*]quinolines **7** and **8** could result in interesting features in terms of selectivity for specific nAChR subunits, Panda et al. did not investigate the biological properties of these two nicotine derivatives, nor have subsequent studies been reported so far.

In this first section we showed a significant number of 2,3-annulated tricyclic conformationally constrained nicotine analogues. In general, the synthesis of all these compounds appeared quite easily: starting from commercially available compounds, only a limited number of steps (ranging from 4 to 7) were needed to obtain the final products. With a few exceptions, product yields of these reactions were typically from modest to excellent, and several catalytic steps have been employed (involving homogeneous acid catalysts or heterogeneous metal systems). For some of these compounds, a detailed biological investigation has been reported; in particular, the nicotine analogue **4** showed from modest to good affinity for both α4β2 and α7 nAChR subunits, although a significant increase in the binding affinities has generally been observed by *N*-quaternization of the pyridine ring or its complexation with boron species.

### 2.2. Synthesis of 3,4-Annulated Tricyclic Conformationally Constrained Nicotine Analogues

The 3,4-annulated tricyclic derivatives are definitely the most extended class of conformationally constrained nicotine analogues reported in the literature. Among them, we start this section considering derivatives with a five-membered ring annulated on the C3,C4 positions of the pyridine ring.

**Scheme 7:**
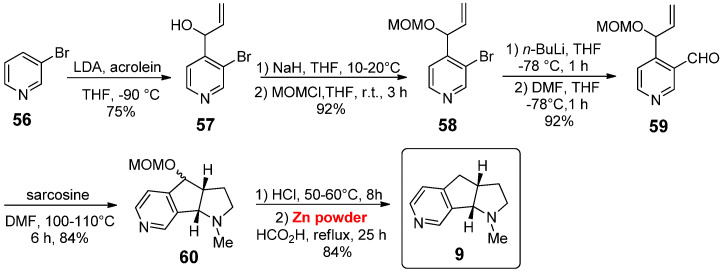
Synthesis of conformationally restricted nicotine analogue **9** developed in 2002 by Zhai et al. [34]. The substoichiometric metal species used as additives are depicted in red.

The synthesis of the hexahydropyrrolo[3,2-*f*]pyrindine **9** has been widely investigated. In 2002, Zhai et al. reported the first synthetic path starting from 3-bromopyridine **56** (Scheme 7) [34]. *Ortho*-lithiation of **56** with LDA and treatment with acrolein gave the alcohol **57** in 75% yield, which was protected by the addition of MOM protecting group to give **58**. Formylation was carried out by treatment with *n*-BuLi at −78 °C and quenching with DMF, affording aldehyde **59** in excellent yields (92%). The subsequent reaction of **59** with sarcosine at 100–110 °C in DMF afforded an azomethine ylide, which then gave tandem [3+2] intramolecular cycloaddition reaction with the formal enal moiety with the formation of hexahydropyrrolo[3,2-*f*]pyrindine tricyclic scaffold **60** in 84% yield and diastereomeric mixture 1: 1.37. Compared to many other [3+2] cycloaddition reactions, in this case no catalytic species was used, because the reaction was simply thermally activated. The final step of this protocol, i.e., a reductive dihydroxylation of **60** with refluxing formic acid, required instead the presence of zinc powder as a substoichiometric (0.74 equiv.) metal additive; working under the same conditions, but in the absence of Zn, no reaction occurred. After this step, the desired analogue **9** was obtained in 84% yield. The same synthesis was reported by the same authors in a following publication, where a more extended number of conformationally restricted nicotine analogues has been synthesized [35].

**Scheme 8:**
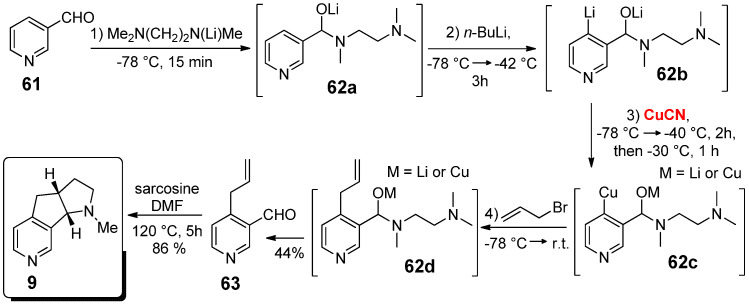
Synthesis of conformationally restricted nicotine analogue **9** developed in 2004 by Zhai et al. [36]. The stoichiometric metal species used as crucial additives are depicted in red.

Interestingly, in 2004 the same group developed an easier strategy for the synthesis of the same hexahydropyrrolo[3,2-*f*]pyrindine **9** from commercially available nicotinaldehyde **61** (Scheme 8) [36]. The first step was the preparation of 4-allyl-nicotinaldehyde **63** through a tandem sequence, consisting of one-pot protection of the aldehyde group with LTMDA (i.e., Me_2_N(CH_2_)_2_N(Li)Me), *ortho*-lithiation with *n*-BuLi, formation of the corresponding cuprate by addition of 2.1 equiv. of CuCN as a metal species, allylation with allyl bromide and final deprotection. It is worth emphasizing that all these steps were carried out in a one-pot fashion without isolating intermediates **62a**–**d**, thus directly obtaining the allylated product **63**. Then, the reaction with sarcosine at 120 °C in DMF afforded an azomethine ylide, which then gave a tandem thermal [3+2] intramolecular cycloaddition with the formal enal moiety, thus generating the final tricyclic nicotine analogue **9** in good yield (86%). In this new protocol of Zhai et al., the cuprate formation by using stoichiometric amount of CuCN as a metal species plays a crucial role in avoiding significant amounts of polyalkylation by-products.

All the above-mentioned studies of Zhai et al. were focused only on the synthesis of the nicotine analogue **9**. To the best of our knowledge, a biological study on this compound was only reported in 2013 by Yin and co-workers; interestingly, they showed a very high affinity of **9** with the [^3^H]-cytisine binding sites in SH-EP1-α4β2-nAChR cell membranes, as testified by a binding affinity constant K_i_ = 21.5 nM [37]. In the same study, **9** also showed promising pharmacological effects on transgenic Alzheimer’s disease mice. 

**Scheme 9:**
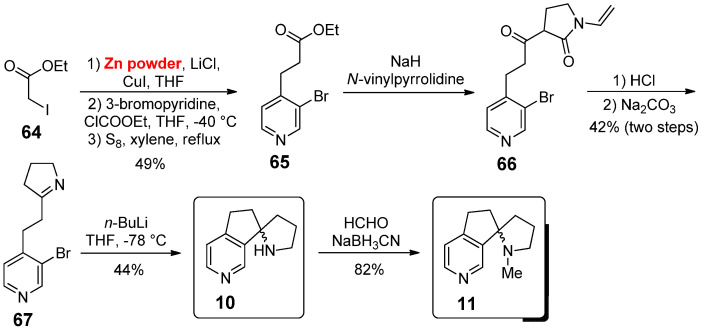
Synthesis of conformationally restricted spiro-annulated nicotine analogue **10** and **11** developed in 2002 by Ullrich et al. [38]. The stoichiometric metal species used as additives are depicted in red.

To the same subclass of 3,4-annulated tricyclic conformationally constrained nicotine analogues also belong spiro-annulated derivatives **10** and **11**, whose synthesis was described in 2002 by Ullrich et al. (Scheme 9) [38]. In this case, the starting point was ethyl 2-iodoacetate **64**; after conversion into organocopper by treatment with CuI in the presence of activated Zn powder as a stoichiometric additive, a Michael-conjugated addition to 3-bromopyridinium ethyl formate and subsequent aromatization in the presence of elemental sulfur gave **65** in 49% yield. Its reaction with *N*-vinylpyrrolidone (in the presence of NaH) afforded β-ketolactam **66**; under acid conditions, it was directly subjected to the removal of the vinyl group, lactam hydrolysis and decarboxylation, yielding a γ-aminoketone that finally cyclized in the presence of Na_2_CO_3_, giving imine **67**. The spiro-annulated nicotine analogue **10** was obtained in 44% yield by reaction of **67** with *n*-BuLi at −78 °C, while its *N*-methylation with formaldehyde and NaBH_3_CN, following an Eschweiler–Clarke protocol, afforded the spiro-annulated nicotine analogue **11** in 82% yield.

In the same investigation, Ullrich et al. also evaluated the biological activity of the two spiro-annulated nicotine analogues **10** and **11**; these compounds were optically resolved and each enantiomer was then evaluated for its ability to displace [^3^H]-cytisine in a rat forebrain preparation. In particular, (+)-**10** bound at K_i_ = 53.1 nM with a 10-fold higher affinity than its enantiomer (–)-**10** (K_i_ = 533 nM); moreover, the introduction of the *N*-methyl substituent resulted in a significant improvement, probably due to the gain of fortified receptor interaction; (+)-**11** (K_i_ = 4.79 nM) appeared to be the most interesting ligand of this set as it not only bound in the low nanomolar range but also exhibited a 30-fold higher affinity than its enantiomer (–)-**11** (K_i_ = 148 nM). Therefore, a combination of the conformational restriction with the beneficial effects of the *N*-methyl group made nicotine analogue (+)-**11** a potential candidate for further investigation, such as peripheral binding and ntinociceptive behavior, which may result in a new lead structure for the development of novel nAChR agonists.

**Scheme 10:**
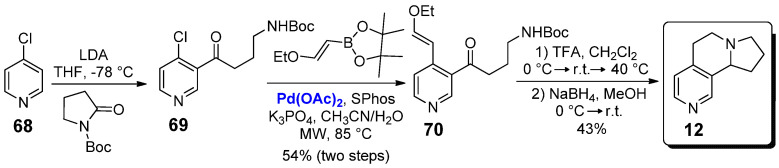
Synthesis of conformationally restricted nicotine analogue **12** reported in 2012 by Kristensen and co-workers [39]. The catalytic species involved in the synthetic pathway are indicated in blue.

Regarding 3,4-annulated tricyclic nicotine analogues with a six-membered ring fused on the C3,C4 positions of the pyridine ring, a very appealing system is hexahydropyrrolo[2,1-*a*][2,7]naphthyridine **12**. The first synthetic pathway was described in 2012 by Kristensen and co-workers (Scheme 10) [39]. The 4-chloropyridine **68** was *ortho*-lithiated and treated with pyrrolidinone to give **69**, which was then used in the following catalytic step: a Suzuki–Miyaura cross-coupling reaction with a vinyl boronic ester, performed with Pd(OAc)_2_ (3 mol%) as the catalyst, at 85 °C for 70 min under microwaves, to give 3,4-disubstituted pyridine **70** in 54% yield. The subsequent treatment with TFA/CH_2_Cl_2_ (1:1 *v/v*), which allowed the formation of the tricyclic scaffolds, followed by one-pot reduction with NaBH_4_, finally gave the racemic hexahydropyrrolo[2,1-*a*][2]naphthyridine **12** in 43% yield.

**Scheme 11:**
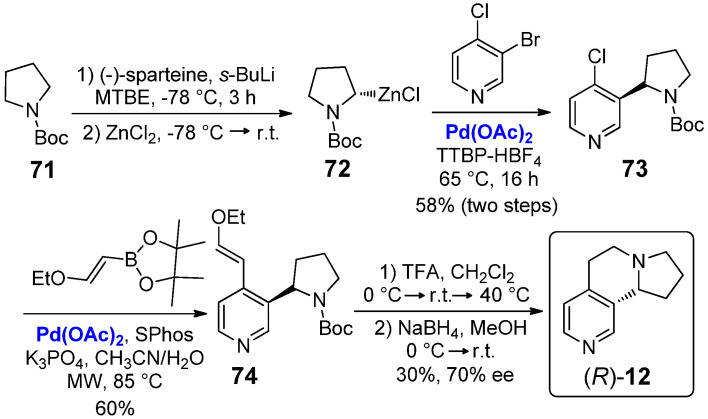
Synthesis of conformationally restricted nicotine analogue (*R*)-**12** reported in 2015 by Kristensen and co-workers [21]. The catalytic species involved in the synthetic pathway are indicated in blue.

Such synthetic path allowed for the preparation of nicotine analogue **12** in racemic form. In a more recent study, the same research group instead proposed an appealing enantioselective synthesis of (*R*)-**12** (Scheme 11) [21]. First of all, *N*-Boc pyrrolidine **71** was lithiated with stoichiometric amounts of (–)-sparteine/*s*-BuLi, followed by transmetalation with 1.0 equiv. of ZnCl_2_, to give optically active intermediate **72**. A Palladium-catalyzed Negishi coupling with 3-bromo-4-chloropyridine at 65 °C, performed in the presence of Pd(OAc)_2_ (5 mol%), afforded the α-arylpyrrolidine **73** in 58% yield. Then, a Suzuki–Miyaura coupling of **73** with a vinyl boronic ester, performed with 3 mol% of Pd(OAc)_2_, afforded the optically active product **74**, followed by treatment with TFA/CH_2_Cl_2_ (1:1 *v/v*) and subsequent one-pot reduction with NaBH_4_ to give the final (*R*)-**12** in 30% yield and 70% ee.

In the same study, Kristensen and co-workers also reported a detailed investigation of the biological activity of (*R*)-**12**; in vitro tests showed that it is a potent and selective antagonist of the α4β2-subtype of the nAChRs in whole cell of *Xenopus laevis* oocytes, attributed to the conformational restriction of the ethylene bridge, as well as to the absolute stereochemistry. In particular, studies were carried out on both receptor stoichiometries (α4)_3_(β2)_2_ and (α4)_2_(β2)_3_; (*R*)-**12** inhibited maximal acetylcholine (1 mM) in (α4)_3_(β2)_2_ nAChR with IC_50_ of 135 μM (K_i_ = 15 μM), while in (α4)_2_(β2)_3_ nAChR with IC_50_ of 87 μM (K_i_ = 0.34 μM). Preliminary in vivo investigations of the antidepressant activity of (*R*)-**12** in the mice’s forced swim test were also carried out, but no effects on swimming were found at the tested doses.

**Scheme 12:**
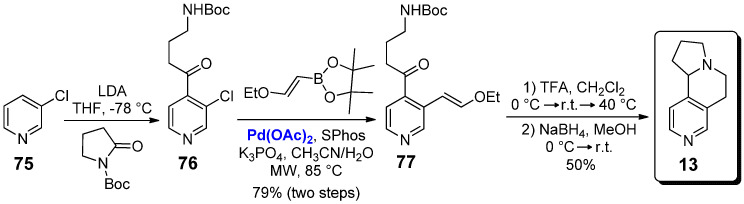
Synthesis of conformationally restricted nicotine analogue **13** reported in 2012 by Kristensen and co-workers [39]. The catalytic species involved in the synthetic pathway are indicated in blue.

The hexahydropyrrolo[2,1-*a*][2,6]naphthyridine **13** is structurally related to the previous nicotine derivative **12**; in fact, its synthesis was reported in 2012 by Kristensen and co-workers following the same three-steps pathway described in Scheme 10 for **12**, but starting from the commercially available 3-chloropyridine **75** as the reagent (Scheme 12) [39]. Unfortunately, for this specific compound no biological studies have been reported to date.

Among all the conformationally restricted derivatives of nicotine, the 1*H*-pyrrolo[3,2-*h*] isoquinoline **14** and its corresponding *N*-methylated analogue **15** are without any doubt the most widely synthesized and investigated.

**Scheme 13:**
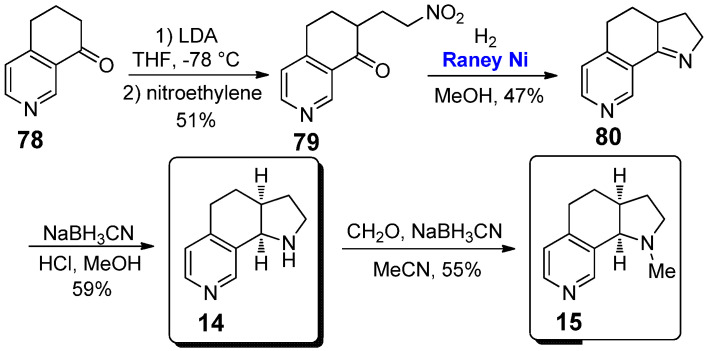
Synthesis of conformationally restricted nicotine analogues **14** and **15** developed in 1993 by Glassco et al. [40]. The catalytic species involved in the synthetic pathway are indicated in blue.

The original synthetic pathway, allowing for the preparation of both these compounds, has been reported in 1993 by Glassco et al., starting from 6,7-dihydroisoquinolin-8(5*H*)-one **78** (Scheme 13) [40]. Interestingly, this procedure was very similar to that reported in 1983 by Seeman and co-workers for the synthesis of the nicotine analogue **4** (see Scheme 2) [22], involving: (a) a Michael addition on nitroethylene of the lithium enolate of ketone **78**; (b) the reduction of the nitro group of **79** to amine with H_2_ in the presence of heterogeneous Raney nickel catalyst (0.35 mol%), followed by intramolecular condensation to afford cyclic imine **80**; and (c) the reduction of imine **80** with NaBH_3_CN, giving the final 1*H*-pyrrolo[3,2-*h*] isoquinoline **14** in 59% yield. The methylation of **14** using formaldehyde and NaBH_3_CN afforded instead the corresponding analogue **15** in 55% yield. Concerning the catalytic step, it is worth emphasizing that the metal catalyst was removed by simple filtration on a Celite pad, confirming once again the advantages of working with heterogeneous and recoverable metal catalysts [41,42,43]. Such a strategy has been followed for many years for the preparation of both pyrrolo[3,2-*h*] isoquinolines **14** and **15**, as reported in 2002 by Crooks et al. [23], or for the synthesis of the only pyrrolo[3,2-*h*]isoquinoline **14**, as reported in 1998 by Vernier et al. [44] and in 2004 by Janda et al. [24].

**Scheme 14:**
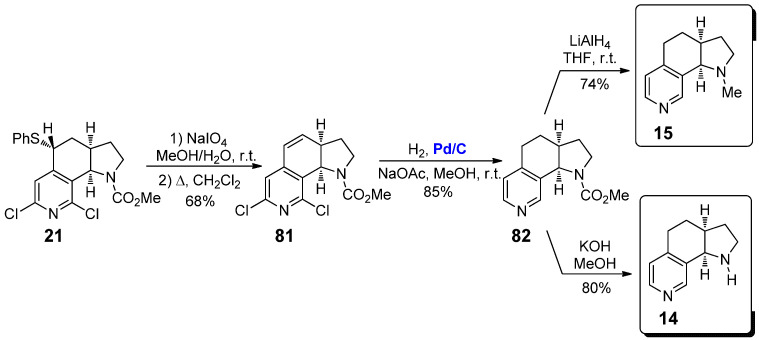
Synthesis of conformationally restricted nicotine analogues **14** and **15** developed in 2004 by Sarkar et al. [45]. The catalytic species involved in the synthetic pathway are indicated in blue.

However, more recently other synthetic strategies for the preparation of nicotine derivatives **14** and, above all, **15,** have been successfully developed. An appealing strategy has been described in 2004 by Sarkar and co-workers, starting from the conformationally constrained nicotine derivative **21** (Scheme 14), whose synthesis will be described below in the same Section 2.2 [45]. First of all, **21** was converted into alkene **81** by oxidation of the thioether moiety with NaIO_4_ and following *syn*-elimination of the resulting sulfoxide. The concomitant double bond reduction and dechlorination was performed by catalytic hydrogenation with H_2_, in the presence of 10 wt.% Pd/C as the catalyst and sodium ascorbate as additive, affording **82** in 85% yield. From **82** it was possible to obtain both pyrrolo[3,2-*h*] isoquinolines **14** and **15**: on the one hand, treatment with LiAlH_4_ in diethyl ether at room temperature afforded **15** in 74% yield; on the other, the reaction with refluxing KOH in methanol afforded the corresponding NH-free analogue **14** in 80% yield.

**Scheme 15:**
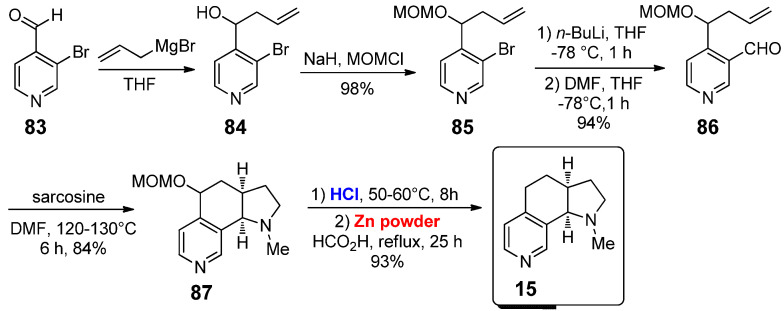
Synthesis of conformationally restricted nicotine analogue **15** developed in 2006 by Zhai and co-workers [35]. The catalytic species involved in the synthetic pathway are indicated in blue, and the stoichiometric metal species used as additives are depicted in red.

A further synthetic strategy for the preparation of pyrrolo[3,2-*h*] isoquinoline **15** has been described in 2006 by Zhai and co-workers (Scheme 15) [35]. The starting point was the addition of allyl Grignard to 3-bromo-4-carboxaldehyde **83** in THF as the solvent, which afforded alcohol **84**. The subsequent MOM-protection and formylation sequence afforded aldehyde **86** in 94% yield. The tricyclic compound **87** was obtained by a metal-free, thermally promoted (120–130 °C) intramolecular [3+2] cycloaddition of a formal enal moiety of **86** with an azomethine ylide generated in situ by the addition of sarcosine. Only the last step involved catalytic processes and stoichiometric metal species as additives; catalytic amounts of hydrochloric acid were successfully used for the MOM-deprotection, followed by the reduction of resulting alcohol with refluxing formic acid, which instead required the presence of zinc powder as a metal additive. In this way, the final nicotine analogue **15** was obtained in 93% yield.

For the sake of completeness, it is worth emphasizing that in 2011 a further synthetic procedure for the preparation of the conformationally restricted nicotine analogue **15** has been reported by Crooks and co-workers, starting from 3-bromo-4-methylpyridine, although it is out of the scope of our mini-review because it did not involve any catalytic step [25].

Several biological studies have been carried out on both pyrrolo[3,2-*h*]isoquinolines **14** and **15** by Glassco et al. The first pharmacological studies were reported in 1993; both compounds were resolved into their enantiomers and tested for their ability to decrease spontaneous activity and inhibition of the tail-flick response (antinociception) in mice, obtaining the best results for compound (+)-**15** [40]. Antagonism studies in mouse brain tissue with mecamylamine were also carried out; in particular, (–)-**15** exhibited low affinity for nicotine binding with K_i_ = 605 ± 217 nM, while failed to displace binding even at 10 μM; racemic **14** competed for binding with a K_i_ = 167 ± 19 nM. In 1996, a more extended study by the same group showed an extensive examination of both enantiomers of the nicotine analogue **15** in drug discrimination, discovering that neither generated nicotine-like responses up to doses that suppress rate of responding; these results clearly demonstrated for the first time that a potent antinociception can be produced with nicotine analogs that do not bind to nicotine receptors [46]. Further in vivo and in vitro studies were performed in 1997 by the same authors, who studied the inhibition of the binding of [^3^H]-*L*-nicotine and [^125^I]-α-BGTX to mouse brain thalamic membranes by (+)-**15** and (–)-**15**, as well as by (+)-**1** and (–)-**1** used for comparison [47]. Concerning the inhibition of [^3^H]-*L*-nicotine, (–)-**1** was the most potent inhibitor (K_i_ = 3.1 nM), 30-fold more potent than (+)-**1**(K_i_ = 99 nM); (–)-**15** (K_i_ = 23 mM) and (+)-**15** (K_i_ = 39 mM) were much less potent. For the inhibition of [^125^I]-α-BGTX, both (–)-**15** (K_i_ = 0.98 mM) and (+)-**15** (K_i_ = 6.2 mM) were more potent inhibitors, which was nearly identical to that of (+)-**1**. The occurrence for **15** of some nicotine-like pharmacological effects was therefore demonstrated, although authors excluded that the strong agonist effects could be mediated by the α4β2 or α7 subtypes of nAChRs, and other nicotinic receptor subtypes remain as possible candidates. Moreover, the *N*-methylated pyrrolo[3,2-*h*] isoquinoline **15** was also found by Glassco et al. as a promising agonists for the treatment of cognitive dysfunction such as is seen in Alzheimer’s disease [48].

However, other groups also investigated the biological features of pyrrolo[3,2-*h*] isoquinolines **14** and **15**. In 1998, Vernier et al. reported the ability of nicotine analogues, including **14**, to increase [Ca^2+^] levels in HEK cells stably transfected with human nAChR subunits α2β4, α3β4, α4β4, α3β2 and α4β2 [44]. Crooks et al. reported for these two compounds only a modest binding affinity towards the α4β2 and the α7 nAChRs, which is very low if compared to the above mentioned nicotine analogue **4**, even in the case of further complexation of the nitrogen atom of the pyridine scaffold with boron species [23,26,28], while *N*-quaternization of the pyridine scaffold of **14** and **15** could easily eliminate inhibitory activity at both α4β2* and α7* nAChRs, affording high affinity and selectivity for the nAChRs mediating nicotine-evoked dopamine release from striatum [27]. More recently, Wang, Zhang et al. discovered that *N*-methylated pyrrolo[3,2-*h*] isoquinoline **15** was a potential candidate as a human nAChR α7 agonists for the clinical treatment of the schizophrenia cognitive disorders [49].

**Scheme 16:**
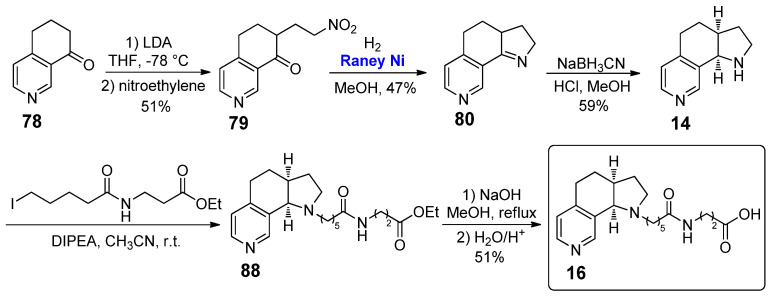
Synthesis of conformationally restricted nicotine analogue **16** developed in 2003 by Janda et al. [29]. The catalytic species involved in the synthetic pathway are indicated in blue.

The pyrrolo[3,2-*h*] isoquinoline **16**, bearing a different chain attached to the nitrogen atom of the pyrrolidine moiety, was easily synthesized in 2003 by Janda et al. using a procedure very similar to that of Glassco et al. for the preparation of analogue **15** (Scheme 16) [29]. After obtaining amine-free compound **14**, it was treated in the presence of DIPEA as a base with the corresponding iodide of the desired linker to give the ester **88**, which was then hydrolyzed to the final nicotine derivative **16** by saponification with NaOH in refluxing MeOH.

As we already described in Section 2.1 for the nicotine derivative **5**, compound **16** was also used by Janda et al. as a constrained nicotine hapten, coupled to the carrier protein KLH in order to study its immunogenic effects for the possible development of nicotine vaccines; immunization of mice using a serum based on **16** resulted in high antibody titers (~25,000) and affinity for (*S*)-nicotine (K_d_ 1.0 ± 0.10 µM), nearly 3-fold improved with respect to their previous generation of serum based on other conformationally free nicotine analogues. The biological interest of pyrrolo[3,2-*h*] isoquinoline **16** was further confirmed by its involvement in some patents focused on the development of such vaccines [30,31].

**Scheme 17:**
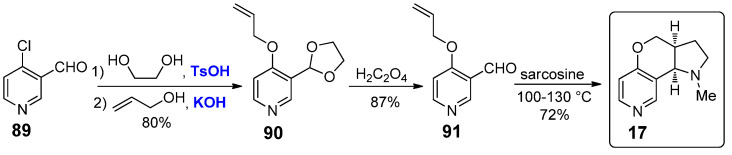
Synthesis of conformationally restricted nicotine analogue **17** developed in 2006 by Zhai and co-workers [35]. The catalytic species involved in the synthetic pathway are indicated in blue.

The pyrrolo[2’,3’:4,5]pyrano[3,2-*c*]pyridine **17** is a 3,4-annulated tricyclic nicotine analogue with an oxygen-containing six-membered ring fused on the C3,C4 positions of the pyridine scaffold. Its synthesis has been reported in 2006 by Zhai and co-workers, starting from 4-chloronicotinaldehyde **89** (Scheme 17) [35]. The –CHO protection of **89** as dioxolane was carried out by treatment with 1,2-ethanediol under catalytic amounts (0.17 mol%) of TsOH, followed by the reaction with allyl alcohol under basic catalysis to obtain allyl ether **90**. The cleavage of the acetal group of **90** was performed in the presence of oxalic acid, allowing for a cleaner reaction product **91** in comparison with other protic acids. The last step was a metal-free, thermally-promoted intramolecular [3+2] cycloaddition induced by treatment with sarcosine, affording final nicotine derivative **17** in 72% yield. Despite its high synthetic interest, to the best of our knowledge no biological studies have been reported to date on such a compound.

**Scheme 18:**
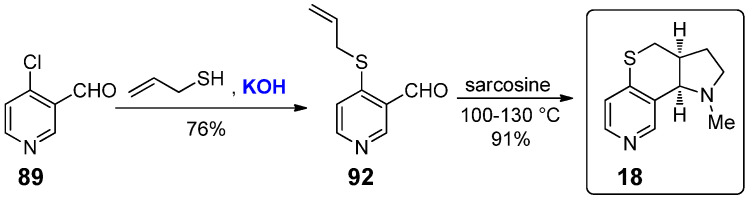
Synthesis of conformationally restricted nicotine analogue **18** developed in 2006 by Zhai and co-workers [35]. The catalytic species involved in the synthetic pathway are indicated in blue.

The pyrrolo[2’,3’:4,5]thiopyrano[3,2-*c*]pyridine **18** differs from **17** only because of the presence of a sulfur atom instead of an oxygen one. The synthesis, reported in 2006 by Zhai et al., started again from 4-chloronicotinaldehyde **89** (Scheme 18) [35]; the protection–deprotection strategy of the aldehyde moiety was not required in this case, therefore **89** was directly treated with allyl thiol under basic catalysis, providing thioether **92**. At this point, the same metal-free, thermally-promoted intramolecular [3+2] cycloaddition induced by the treatment with sarcosine afforded nicotine derivative **18** in 91% yield. Similarly to the above-mentioned oxygenated analogue **17**, no biological studies for compound **18** have been conducted.

**Scheme 19:**
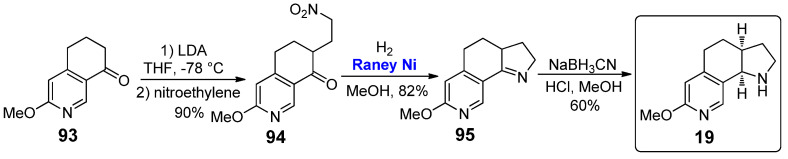
Synthesis of conformationally restricted nicotine analogue **19** developed in 1998 by Vernier et al. [44]. The catalytic species involved in the synthetic pathway are indicated in blue.

Proceeding forward, regarding the 3,4-annulated tricyclic conformationally constrained analogues bearing substituents on the pyridine scaffold, the pyrrolo[3,2-*h*] isoquinoline **19** is characterized by the presence of a –OMe group on the C6 position of the pyridine scaffold. The preparation of such a compound, reported in 1998 by Vernier et al., was very similar to that reported in 1993 for the preparation of **15** by Glassco et al. (see Scheme 13), although starting from 3-methoxy-6,7-dihydroisoquinolin-8(5*H*)-one **93** (Scheme 19) [44].

Vernier et al. also reported some biological tests of this nicotine analogue, studying the increase in [Ca^2+^] levels in HEK cells stably transfected with several human nAChR subunits, observing high values in the case of α2β4 (EC_50_ = 87 ± 7 μM, efficacy 22% ± 4 normalized to the response of acetylcholine or nicotine) and α4β4 (EC_50_ = 32 ± 13 μM, efficacy 44% ± 4 normalized to the response of acetylcholine or nicotine). However, a more appealing investigation was reported in 2003 by Rao and co-workers; the conformationally constrained nicotine derivative **19** was found to selectively activate α2β4 and α4β4 human recombinant neuronal nAChRs (K_i_ values displacing the binding of [^3^H]-nicotine and [^3^H]- quinuclinidylbenzilate were 1.0 ± 0.3 μM and 2.6 ± 0.3 μM, respectively) with no activation of α4β2, the presumed high-affinity nAChRs in rodent brain [50]. Moreover, it is worth emphasizing that pyrrolo[3,2-*h*] isoquinoline **19** has also found application in some patents, focused on the treatment of inflammation and autoimmunity in the central nervous system [51], on improving tear production for treatment of dry eye syndrome [52], and on a transdermal therapeutic system [53].

**Scheme 20:**
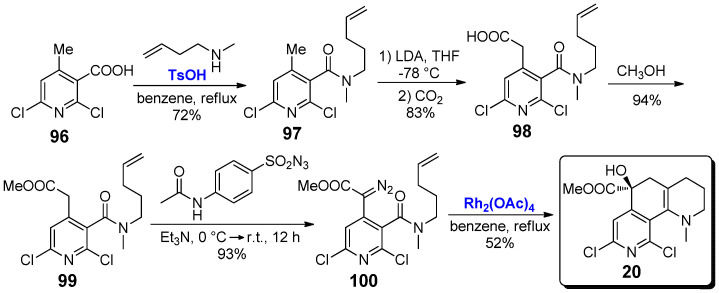
Synthesis of conformationally restricted nicotine analogue **20** developed in 2000 by Sarkar and co-workers [54]. The catalytic species involved in the synthetic pathway are indicated in blue.

The conformationally restricted nicotine analogue **20** is characterized by a more complex chemical structure, bearing two chlorine atoms on the C1 and C6 positions of pyridine scaffold. The synthesis of this compound was reported in 2000 by Sarkar and co-workers starting from 2,6-dichloro-4-methylnicotinic acid **96** (Scheme 20) [54]. First of all, treatment of **96** with *N*-methylpent-4-enamine in the presence of catalytic amounts of TsOH afforded amide **97** in 72% yield, which was then subjected to a C1-homologation. The resulting carboxylic acid **98**, obtained in high yield (83%) was converted into methyl ester **99**, then it was subjected to a diazotransfer reaction to give diazoacetic ester **100**. The final step was an intramolecular Hamaguchi–Ibata reaction, carried out thanks to the presence of Rh_2_(OAc)_4_ (1 mol%) as catalyst for 1 h in refluxing benzene, which afforded nicotine analogue **20** in 52% yield. The same path was re-proposed in 2003 by Sarkar and co-workers in a more extended investigation, where **20** was synthesized together with other nicotine derivatives [55]. However, despite its significant interest from a merely synthetic point of view, no studies about biological activity have been reported to date.

**Scheme 21:**
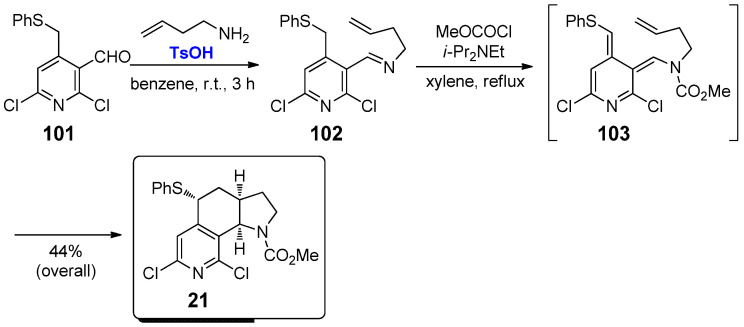
Synthesis of conformationally restricted nicotine analogue **21** developed in 2004 by Sarkar et al. [45]. The catalytic species involved in the synthetic pathway are indicated in blue.

Another interesting 3,4-annulated tricyclic conformationally constrained nicotine derivative bearing two chlorine atoms on the C1 and C6 positions of pyridine scaffold is the compound **21**, synthesized in 2004 by Sarkar et al. by a two steps procedure (Scheme 21) [45]. The first one involved a catalytic process, where aldehyde **101** was treated with homoallyl amine in presence of TsOH as acid catalyst (0.1 mol%) to give imine **102**. The second step involved the use of methyl chloroformate in the presence of DIPEA; under these conditions, the intermediate **103** was generated through a formal imine tautomerisation and intramolecular trapping, which then evolved to the final tricyclic nicotine analogue **21** (44% yield) via intramolecular Diels–Alder reaction.

The biological activity of the nicotine analogue **21** was investigated only in one study of Wainer and co-workers; liquid chromatography columns containing stationary phases based on immobilized α3β4 and α4β2 nAChR subtypes were used to screen a series of nicotine derivatives, including conformationally constrained analogue **21**. In particular, they found that this derivative should be active at the α4β2 nAChR but not at the α3β4 nAChR (Δml values, calculated as breakthrough volume of epibatidine alone minus the breakthrough volume of epibatidine after the addition of **21** on the nAChR immobilized-columns, were 0.17 and −0.04 for α4β2 and α3β4, respectively) [56].

**Scheme 22:**
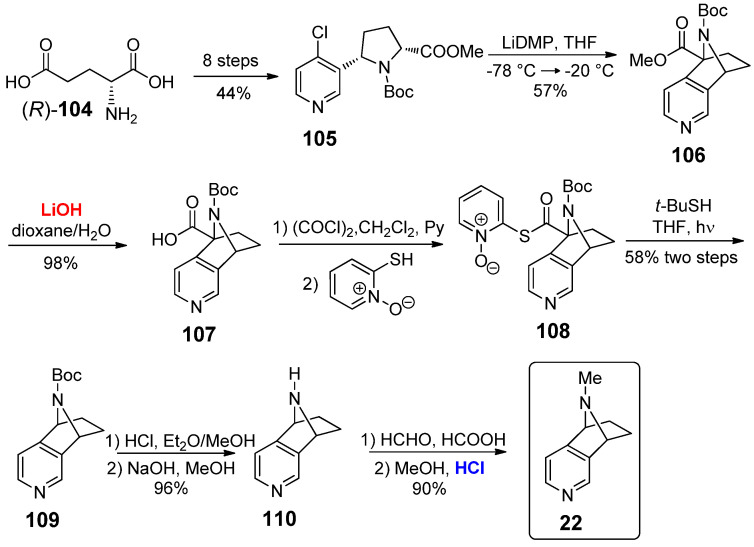
Synthesis of conformationally restricted nicotine analogue **22** developed in 2001 by Rapoport and co-workers [57]. The catalytic species involved in the synthetic pathway are indicated in blue, and sub-stoichiometric metal species used as additives are depicted in red.

Some examples of 3,4-annulated tricyclic nicotine analogues with a bridged structure fused on the C3,C4 positions of the pyridine scaffold have also been reported. The azabicyclo[2.2.1]heptano[2,3-*c*]pyridine **22** is an appealing scaffold, whose preparation was described in 2001 by Rapoport and co-workers (Scheme 22) [57]. The starting point was *D*-glutamic acid (*R*)-**104**, converted into proline derivative **105** through an eight-steps strategy. The subsequent cyclization of **105** into the bridged derivative **106** was promoted by treatment with lithium 2,2,6,6-tetramethylpiperidide (LiDMP) at a low temperature; the methyl ester group of **106** was converted into acid **107** in a water–dioxane solvent mixture, in the presence of substoichometric amounts of LiOH as additive. The –COOH group of **107** was removed by a two-steps procedure, consisting in the condensation with 2-mercaptopyridine *N*-oxide to give thioester **108**, followed by irradiation with a 100 W incandescent lamp in the presence of *t*-BuSH, which gave **109** in 56% yield. The removal of the Boc protecting group under acid conditions afforded the NH-free bridged nicotine derivative **110**, which was finally methylated to give azabicyclo[2.2.1]heptano[2,3-*c*]pyridine **22** in 90% yield.

Although a potential biological application of nicotine derivative **22** has been postulated in 2004 by Carroll et al. [58], only in 2007 they actually reported the inhibition of radioligands binding for the α4β2 and α7 nAChRs (using [^3^H]-epibatidine and [^125^I]-iodoMLA, respectively), but modest results were obtained in comparison to other nicotine derivatives (<15% inhibition at 31.6 uM for α4β2 [^3^H]-epibatidine; 0% inhibition at 50 nM for α7 [^125^I]-iodoMLA) [59].

**Scheme 23:**
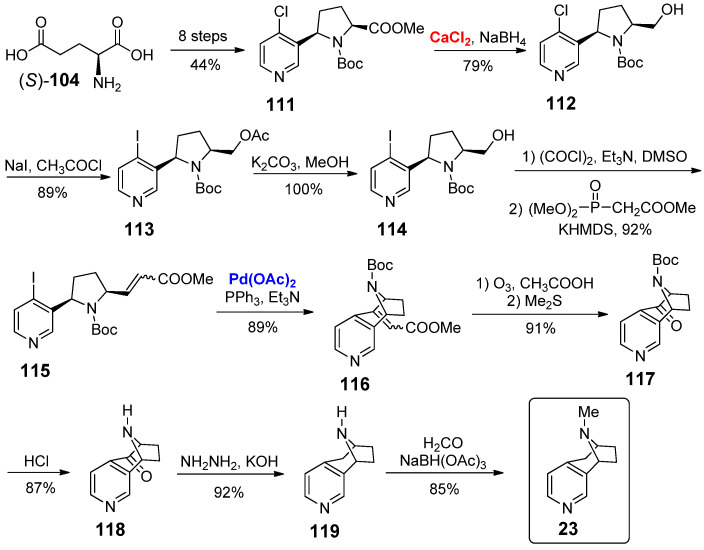
Synthesis of conformationally restricted nicotine analogue **23** developed in 2000 by Rapoport and co-workers [60]. The catalytic species involved in the synthetic pathway are indicated in blue, while stoichiometric metal species used as additives are depicted in red.

The epiminocyclohepta[*c*]pyridine **23** is a different 3,4-annulated tricyclic nicotine analogue with a bridged structure, whose synthesis has been reported in 2000 by Rapoport and co-workers (Scheme 23) [60]. In this case the starting point was enantiopure *L*-glutamic acid (*S*)-**104**, i.e*.,* the opposite enantiomer of that used for the preparation of **22**. First of all, (*S*)-**104** was converted into derivative **111** through an eight-steps strategy (44% overall yield). The methyl ester group was then reduced to primary alcohol **112** by treatment with NaBH_4_ in the presence of a stoichiometric amount of CaCl_2_ (1.0 equiv.) as additive. At this point, the chlorine atom of **112** was replaced with an iodine atom through treatment with NaI in the presence of acetyl chloride, affording **113** in 89% yield, which was then deacetylated under basic conditions to give **114**. Moffatt–Swern oxidation of **114**, followed by one-pot treatment with potassium trimethyl phosphonoacetate, provided the olefin **115** in 92% yield, then subjected to an intramolecular Heck coupling with Pd(OAc)_2_ and PPh_3_ as the catalytic system, giving cyclic [3.2.1]homotropane analogue **116** in 89% yield. The ozonolysis to ketone **117**, followed by the removal of Boc protecting group under acid conditions, gave NH-free bridged nicotine derivative **118**. Several reducing conditions were tested for the reduction of ketone **118**, but only the Wolff–Kishner conditions afforded **119** in very high yield (92%). The final methylation step, carried out with H_2_CO in the presence of NaBH(OAc)_3_, afforded the epiminocyclohepta[*c*]pyridine **23** in 85% yield.

Despite the long and challenging synthetic pathway developed for the synthesis of **23**, to the best of our knowledge no biological studies have been reported to date for this conformationally constrained nicotine analogue.

**Scheme 24:**
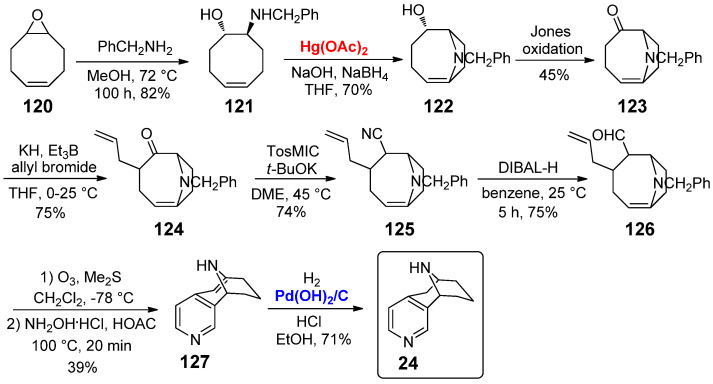
Synthesis of conformationally restricted nicotine analogue **24** developed in 1986 by Kanne et al. [61]. The catalytic species involved in the synthetic pathway are indicated in blue, and stoichiometric metal species used as additives are depicted in red.

However, among all the 3,4-annulated tricyclic nicotine analogue with a bridged structure fused on the C3,C4 positions of the pyridine scaffold, pyrido[3,4]homotropane **24** is definitely the most widely investigated. The synthesis was reported for the first time in 1986 by Kanne et al. (Scheme 24) [61]. The preparation started with the epoxidation of cyclooctadiene to generate monoepoxide **120**, which was then treated with benzylamine giving **121** in good yield (82%). Afterward, a Hg-mediated intramolecular hydroamination afforded bicyclopyrrolidine alcohol **122;** despite the toxicity of Hg(OAc)_2_, its stoichiometric use (1.0 equiv.) was mandatory for achieving the hydroamination step. The Jones oxidation of **122** efficiently produces ketone **123**, which was then α-allylated by treatment with KH and allyl bromide to give **124** in 75% yield. The interconversion of the keto group of **124** into cyano compound **125** was performed using tosylmethyl isocyanide in the presence of *t*-BuOK; then, it was treated with DIBAL-H at −78 °C to give aldehyde **126**. The construction of the pyridine moiety was carried out by ozonolysis of the double bond, followed by reaction with NH_2_OH· HCl in presence of acetic acid. The catalytic process occurred as the last step: debenzylation of **127** was carried out by hydrogenolysis with H_2_ in the presence of the Pearlman’s catalyst Pd(OH)_2_/C, which showed the advantage of working cleanly (owing to the possibility of an easy removal by filtration on dicalite diatomite) and under mild conditions, giving the final derivative **24**. The same synthetic pathway has been followed for many years, as reported in 1988 by the same group [62], and in 2006 by Carroll and co-workers [63]. For the sake of completeness, it is worth emphasizing that two enantioselective versions of the synthesis of **24** have also been reported: in 2008 by Zhai et al. [64] and in 2018 by Wang, Xing et al. [65].

The high interest in the pyrido[3,4]homotropane **24** has been confirmed by a significant number of biological investigations. First of all, Kanne et al. showed that derivative **24** possess three times the toxicological activity (intravenous mouse injection) and 16 times the receptor binding (rat brain membranes) of nornicotine (IC_50_ = 8.0 × 10^−8^ M for **24** vs. IC_50_ = 5.0 × 10^−9^ M for nornicotine) [61], as well as a potent pharmacologic activity and binding affinity to both the electroplax nicotinic cholinergic receptor and brain nicotinic sites (inhibition of [^3^H]-nicotine binding: IC_50_ = 1.0 × 10^−9^ M in *Torpedo* membranes, IC_50_ = 5.0 × 10^−9^ M in rat brain membranes; inhibition of [^3^H]-MCC: IC_50_ = 1.0 × 10^−10^ M in *Torpedo* membranes, IC_50_ = 1.0 × 10^−8^ M in rat brain membranes) [62]. In 2002, Seitz et al. reported for **24** a moderate radioligand ([^3^H]-epibatidine rat brain) binding affinity to (α4)_2_(β2)_3_ nAChR subunits (IC_50_ = 5 nM) [66], while in 2005 Casida and co-workers described a strong binding affinity to the α7 nAChR subunit (K_i_ > 100000 nM, 31% of inhibition), but a modest one to the α4β2 nAChR (K_i_ =1.3 ± 0.2 nM) [67]. Carroll and co-workers emphasized the different roles played by the two different enantiomers of pyrido[3,4]homotropane **24**: (*+*)-**24** was characterized by a binding affinity to α4β2 nAChRs (K_i_ = 1.29 ± 0.18 nM) over 260 times higher than (–)-**24** (K_i_ = 350 ± 160 nM), and comparatively (–)-**24** antagonized nicotine-induced antinociception in the tail-flick assay [63]. In a following investigation, electrophysiological studies with rat nAChRs showed that, on the one hand, (*+*)-**24** was a low efficacy partial agonist selective for α4β2-nAChRs (K_i_ = 3.2 ± 0.4 nM), relative to α3β4-nAChRs (K_i_ = 7.7 ± 0.9 nM) and α7-nAChRs (K_i_ = 0 nM), while on the other (–)-**24** was an antagonist with selectivity for α3β4 (K_i_ = 32 ± 3 nM), relative to α4β2 (K_i_ = 41 ± 4 nM) and α7 (K_i_ = 84 ± 10 nM) nAChRs [68]. The structure–activity relationship (SAR) of nicotine derivative 24 as neuronal nicotinic receptor agonist was also evaluated by theoretical calculations of 3D-QSAR models [69,70].

To conclude this second section of our mini-review, the 3,4-annulated tricyclic derivatives is definitely the most extended class of conformationally constrained nicotine analogues reported in the literature. A huge number of different synthetic procedures have been successfully developed: in some cases (especially for systems with a five- or six-membered ring fused on the C3,C4 positions of the pyridine scaffold), the preparation pathways appeared quite short and easy, consisting of a limited number of steps, which also involved catalytic systems or at least (sub)stoichiometric metal species as additives; in other cases (in particular for analogues bearing several substituents on the pyridine scaffold or with a bridged structure fused on the C3,C4 positions of the pyridine scaffold), the number of steps was typically higher, thus resulting in low overall yields of the final *N*-heterocyclic products. Moreover, the large number of biological studies cited in this section also confirmed that this class of conformationally constrained analogues of nicotine is the most investigated in the context of the activity for specific nAChR subunits.

### 2.3. Synthesis of Tetracyclic Conformationally Constrained Nicotine Analogues

**Scheme 25:**
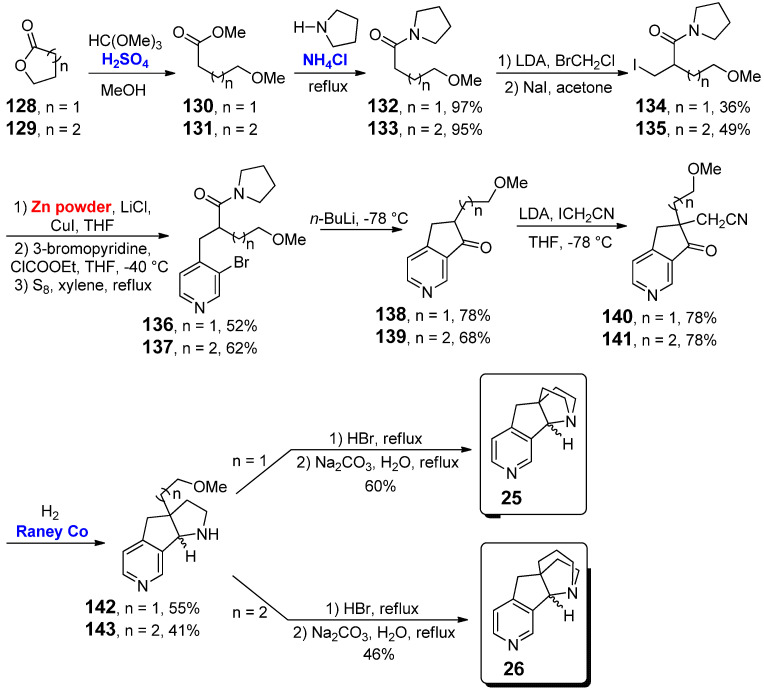
Synthesis of conformationally restricted nicotine analogues **25** and **26** developed in 2002 by Ullrich et al. [38]. The catalytic species involved in the synthetic pathway are indicated in blue, and the stoichiometric metal species used as additives are depicted in red.

Compared to tricyclic derivatives, the number of tetracyclic conformationally restricted nicotine analogues is very limited. To the best of our knowledge, the only two compounds belonging to this class are the nicotine analogues **25** and **26**, whose synthesis was reported in 2002 by Ullrich et al. (Scheme 25) [38]. Methanolysis of γ-butyrolactone **128** and δ-valerolactone **129** in the presence of catalytic amounts of sulfuric acid afforded esters **130** and **131**, respectively, which were then aminolyzed with refluxing pyrrolidine and NH_4_Cl (0.28 mol%), giving amides **132** and **133**. The iodomethyl group was then introduced in the α-position of amide moiety by lithiation of **132**–**133** with LDA, treatment with BrCH_2_Cl and subsequent iodination of chloromethyl-substituted intermediate with NaI. 

Iodides **134**–**135** were converted into the corresponding organocopper systems, used for a Michael addition on the *N*-acylpyridinium salt of the 3-bromopyridine at a low temperature to give the corresponding dihydropyridines; without any isolation, dihydropyridines were directly aromatized with S_8_ in refluxing xylene to give 3-bromo-4-substituted pyridines **136** and **137**. Treatment with *n*-BuLi at −78 °C allowed a metal-halogen exchange of the 3-bromo substituent, followed by intramolecular cyclization to give pyrindinones **138**–**139**, which were then treated with LDA and ICH_2_CN in order to allow the α-substitution of ketone moiety. The resulting **140**–**141** were then subjected to another catalytic process: the hydrogenation of nitriles with H_2_ in the presence of Raney cobalt as efficient, heterogeneous and easily recoverable catalyst, resulting in a cascade reaction that produced annulated pyrrolidines **142** and **143**. The use of Raney Co was due to the fact that the more common Raney nickel gave only modest results. Finally, the last step was the –OMe ether cleavage under acidic conditions (i.e., HBr), followed by thermal heating-promoted cyclization to the azabicyclic ring system under basic conditions, allowing the formation of final tetracyclic nicotine derivatives **25** and **26** in 46–60% yields.

In the same investigation, Ullrich et al. also evaluated the biological activity of these two tetracyclic nicotine analogues **25** and **26**; these compounds were optically resolved and each enantiomer was then evaluated for its ability to binding [^3^H]-cytisine in a rat forebrain preparation. Unfortunately, these analogues bonded in a low micromolar or sub-micromolar range (K_i_ = 962 ± 170 nM for (+)-**25**, 1360 ± 180 nM for (–)-**25**, 318 ± 34 nM for (+)-**26**, 1040 ± 92 nM for (–)-**26**), hypothesizing that the extreme conformational constraint forced their molecular shapes in an unfavorable position, in which binding was not possible. Alternatively, authors supposed that the high steric hindrance of analogues **25** and **26** was not tolerated by the receptors.

To conclude this short section, it is worth emphasizing that the synthetic procedures for the preparation of tetracyclic conformationally restricted nicotine analogues appeared more complicated than those of the previous classes of derivatives described above, mainly due to their complex chemical structures. Some steps definitely worked very well, affording the desired products in high yields; other were instead associated to lower yields, also those involving catalytic species or (sub)stoichiometric metal additives. In our opinion, further investigations should be carried out on this class of compounds, not only from a merely synthetic point of view but also in the context of biological studies.

### 2.4. Synthesis of Other Polycyclic Conformationally Constrained Nicotine Analogues

The last class of nicotine analogues which we will take into account in this mini-review is represented by such systems bearing a bicyclic bridged structure connected (but not fused) to the pyridine scaffold. Although significantly different from all the compounds described so far, these systems can be still considered conformationally restricted analogues of nicotine due to the rigid structure exerted by the bicyclic bridged structure.

**Scheme 26:**
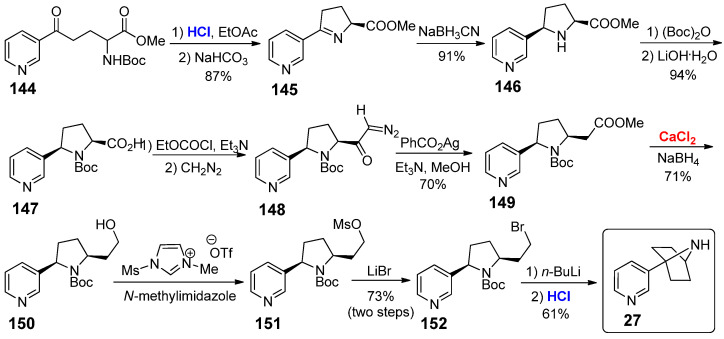
Synthesis of conformationally restricted nicotine analogue **27** developed in 1999 by Rapoport and co-workers [71]. The catalytic species involved in the synthetic pathway are indicated in blue, and the (sub)stoichiometric metal species used as additives are depicted in red.

The first nicotine derivative of this class is the 1-pyridinyl-7-azabicyclo[2.2.1]heptane **27**, whose synthesis was reported in 1999 by Rapoport and co-workers starting from pyridine derivative **144** (Scheme 26) [71]. First of all, the Boc removal was performed under acid catalysis (HCl), followed by intramolecular condensation of the generated amine to give cyclic imine **145**. The reduction of **145** with NaBH_3_CN as reducing agent afforded pyrrolidine **146**; then, the Boc-protection of secondary amine group and subsequent hydrolysis of the ester group afforded **147** in 94% overall yield. Curtius rearrangement and homologation sequence gave compound **149** in 70% yield; the ester group of **149** was successfully reduced to alcohol **150** by treatment of sodium borohydride in the presence of CaCl_2_ (1.0 equiv.) as additive. The use of CaCl_2_ as a stoichiometric metal species was mandatory for obtaining **150** in good yields: authors assumed that the real reducing agent was actually the in situ generated calcium borohydride. Alcohol **150** was converted into bromide **152** in 73% yield passing through mesylate **151** as intermediate. Finally, the desired 1-pyridinyl-7- azabicyclo[2.2.1]heptane **27** was obtained by cyclization of **152** upon treatment with *n*-BuLi, followed by the acid-catalyzed (once again with HCl) deprotection of the *N*-Boc moiety. Despite the high synthetic interest of such nicotine derivative, no biological studies have been reported to date.

**Scheme 27:**
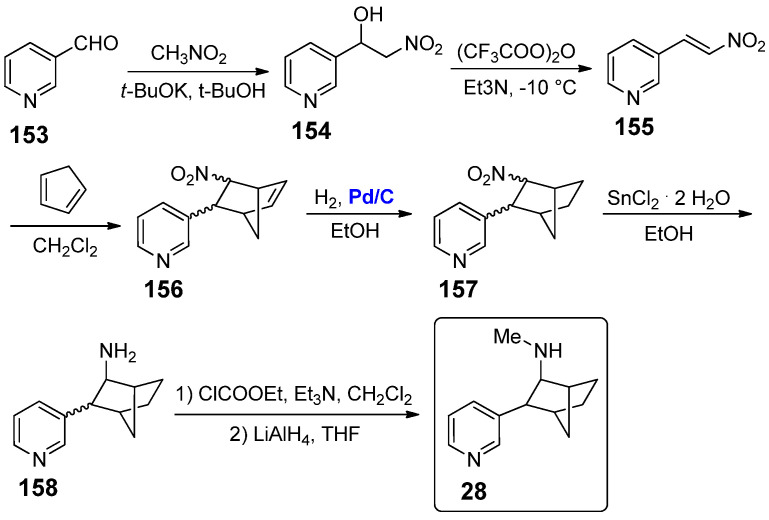
Synthesis of conformationally restricted nicotine analogue **28** developed in 2019 by Manetti and co-workers [72]. The catalytic species involved in the synthetic pathway are indicated in blue.

The introduction of a norbornane moiety as bicyclic bridged structure connected to the pyridine scaffold is a more recent strategy, reported only in 2019 by Manetti and co-workers for the two conformationally restricted nicotine derivatives **28** and **29** [72]. Compound **28** was synthesized from commercially available nicotinaldehyde **153** (Scheme 27); after condensation with nitromethane to give β-hydroxynitro compound **154**, dehydration with (CF_3_COO)_2_O afforded the nitrovinyl pyridine **155**. This intermediate was then subjected to Diels–Alder with cyclopentadiene under thermal conditions, generating norbornene **156**. At this point, the catalytic step of such a procedure occurred, that is, the hydrogenation of norbornene unit of **156** to norbornane **157**, performed in the presence of supported Pd/C catalyst (10 mol% of loading). At the end of the reaction, it was easily removed by simple filtration, confirming once again all the advantages of working with heterogeneous and recoverable palladium catalysts [41,42,43]. The reduction of **157** with SnCl_2_ · 2 H_2_O afforded amine **158**, which was finally methylated to give the nicotine derivative **28**.

**Scheme 28:**
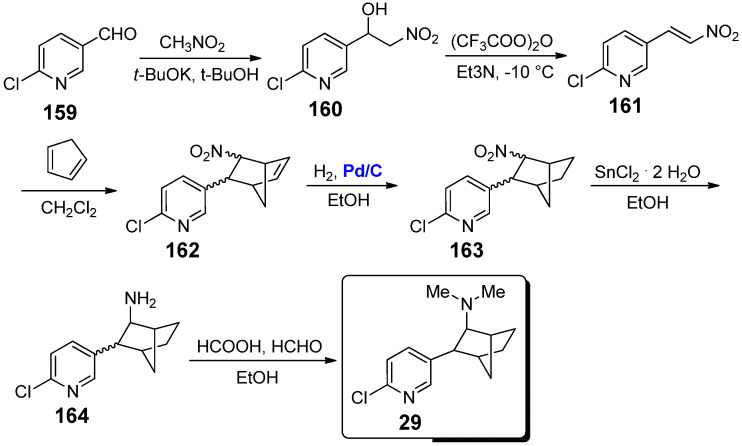
Synthesis of conformationally restricted nicotine analogue **29** developed in 2019 by Manetti and co-workers [72]. The catalytic species involved in the synthetic pathway are indicated in blue.

Compound **29**, differing from **28** by the presence of a chlorine atom on the C6 position of the pyridine scaffold and of a tertiary *N*,*N*-dimethylamino group (instead of a secondary *N*-methylamino) on the norbornane scaffold, was synthesized through a very similar synthetic pathway, using commercially available 6-chloronicotinaldehyde **159** as the starting material (Scheme 28). After nitromethane condensation, dehydratation, Diels–Alder reaction, Palladium-catalyzed hydrogenation and nitro group reduction under the same experimental conditions described above in the Scheme 27, the amine **164** was subjected to a double methylation by Eschweiler–Clarke reaction (carried out in the presence of formaldehyde and formic acid, in absolute ethanol as the solvent), affording the final nicotine derivative **29** in good yields.

In the same study, Manetti and co-workers also reported a detailed biological investigation as nicotinic receptor ligands of these two norbonane-functionalized nicotine derivatives; in fact, they were tested in radioligand binding assays on rat cortex against [^3^H]-cytisine and [^3^H]-methyllycaconitine to measure their affinity for α4β2* and α7* receptors (for the derivative **28**: K_i_ = 10.22 ± 1.09 nM to α4β2*, K_i_ = 352 ± 32 nM to α7*; for the derivative **29**: K_i_ = 43 ± 4 nM to α4β2*, K_i_ > 1000 nM to α7*, 33% inhibition).

In conclusion, nicotine derivatives bearing a bicyclic bridged structure connected to the C3 position of the pyridine scaffold, which can be still considered conformationally restricted analogues due to the rigid structure exerted by the bicyclic bridged structure, showed typically very long synthetic pathways. These compounds represent the last frontier in the context of nicotine derivatives, as well demonstrated by the recent work of Manetti and co-workers, and we are confident that several improvements will be performed in the next year, not only from the synthetic point of view, but also in the frame of biological studies.

## 3. Conclusions

In the context of *N*-heterocyclic compounds [73,74], nicotine is a chiral alkaloid which can target and stimulate nAChRs, with therapeutic potential in the treatment of various neural diseases. However, due to its well-known toxicity for humans, there is considerable interest in the development of synthetic analogues selective for specific nAChR subunits. In this context, conformationally constrained derivatives of nicotine have acquired considerable attention. In this mini-review, we focused the attention on the synthesis of conformationally restricted analogues of nicotine involving one or more catalytic processes. We followed a systematic approach as a function of the heteroarene structure, considering 2,3-annulated tricyclic derivatives (Section 2.1), 3,4-annulated tricyclic derivatives (Section 2.2), tetracyclic derivatives (Section 2.3) and other polycyclic derivatives (Section 2.4). For each of these compounds, although complete synthetic sequences have been described, benefits and drawbacks of steps involving catalytic processes, or at least promoted by (sub)stoichiometric metal species used as additives, have been discussed, as well as some comparative assessments of the various synthetic strategies. For the sake of completeness, for each of these compounds we also briefly discussed the biological studies which have been performed, in particular in the context of their activity for specific nAChR subunits. For an easier and more immediate comparison, all this information has also been briefly summarized in Table 1 below.

The synthetic steps involving catalytic species described above were quite variegated: hydrogenation reactions catalyzed by heterogeneous Raney nickel, cobalt, or carbon- supported palladium catalysts; acid catalyzed hydrolysis or dehydratation involving either inorganic (HCl, HClO_4_, H_2_SO_4_) or organic (TsOH) acids; cross-coupling reactions (in particular, Suzuki–Miyaura coupling) involving homogeneous Pd species; radical mechanism-based reactions initiated by catalytic amounts of a suitable radical initiators (AIBN); and in few cases, also inorganic base catalyzed processes. Similarly, synthetic steps involving (sub)stoichiometric metal species ranged from the in situ formation of organometallic compounds (in the case of zinc powder or CuCN) to the in situ generation of key reagents (for example in the case of CaCl_2_). In our opinion, the catalytic methods for the synthesis of conformationally constrained nicotine derivatives still offers plenty of room for improvements, in particular in the context of the development of more sustainable and green protocols, based on the use of recoverable and recyclable catalysts and additives, possibly coupled with environmental friendly solvents and more efficient energy sources [75,76]. In this context, we are convinced that our mini-review could really help the wide community of synthetic organic chemists to pursue this scope. Structural novelty and medicinal importance of conformationally restricted nicotine analogues will attract synthetic organic chemistry to explore this field in more details in the near future.

## Figures and Tables

**Table 1:** Summary of the literature concerning the synthesis and the biological characterization of conformationally constrained nicotine analogues considered in the present mini-review.

Nicotine Analogue	Synthesis: Starting Material [Ref.]	Biological Studies: Test (Measurement) [Ref.]
**3**	2-methylpyridine-3-carboxaldehyde (**30**) [19]	guinea-pig ileum (EC_50_ = 53 μm for (−)-**3**) [20]
		α4β2 in *X. laevis* (no activity found) [21]
**4**	7,8-dihydroquinolin-5(6*H*)-one (**37**) [22]	rat membranes: α4β2 (K_i_ = 12.2 μM); α7 (K_i_ > 100 μM) [26]
	7,8-dihydroquinolin-5(6*H*)-one (**37**) [23]	striatum: α4β2*, α7* (IC_50_ = 30–310 nM) [27]
	7,8-dihydroquinolin-5(6*H*)-one (**37**) [24]	α7 in *X. laevis* (no agonist activity) [28]
	2-methylpyridine [25]	
**5**	7,8-dihydroquinolin-5(6*H*)-one (**37**) [29]	mice immunization: affinity for (S)-**1** (K_d_ = 0.60 µM) [29]
		patent nicotine vaccines [30]
		patent nicotine vaccines [31]
**6**	2-bromopyridine (**42**) [32]	-
**7**	2-methyl-5-nitronicotinate (**47**) [33]	-
**8**	2-methyl-5-nitronicotinate (**47**) [33]	-
**9**	3-bromopyridine (**56**) [34]	SH-EP1-α4β2 cell membranes (K_i_ = 21.5 nM) [37]
	3-bromopyridine (**56**) [35]	
	nicotinaldehyde (**61**) [36]	
**10**	ethyl 2-iodoacetate (**64**) [38]	α4β2 in rat forebrain (K_i_ = 53.1 nM for (+)-**10**) [38]
**11**	ethyl 2-iodoacetate (**64**) [38]	α4β2 in rat forebrain (K_i_ = 4.79 nM for (+)-**11**) [38]
**12**	4-chloropyridine (**68**) [39]	α4β2 in *X. laevis* ((*R*)-**12**: K_i_ = 0.34 μM, (α4)_2_(β2)_3_) [21]
	*N*-Boc pyrrolidine (**71**) [21]	
**13**	3-chloropyridine (**75**) [39]	-
**14**	6,7-dihydroisoquinolin-8(5*H*)-one (**78**) [40]	mouse brain (K_i_ = 167 ± 19 nM) [40]
	6,7-dihydroisoquinolin-8(5*H*)-one (**78**) [23]	HEK cells with human α2β4, α3β4, α4β4, α3β2, α4β2 [44]
	6,7-dihydroisoquinolin-8(5*H*)-one (**78**) [44]	
	6,7-dihydroisoquinolin-8(5*H*)-one (**78**) [24]	
	nicotine derivative **21** [45]	
**15**	6,7-dihydroisoquinolin-8(5*H*)-one (**78**) [40]	mouse brain ((–)-**15**: K_i_ = 605 ± 217 nM) [40]
	6,7-dihydroisoquinolin-8(5*H*)-one (**78**) [23]	no binding activity to nicotine receptors [46]
	nicotine derivative **21** [45]	[^125^I]-α-BGTX to mouse brain ((+)-**15**: K_i_ = 6.2 mM) [47]
	3-bromo-4-carboxaldehyde (**83**) [35]	*in vivo* treatment of cognitive dysfunction [48]
	3-bromo-4-methylpyridine [25]	clinical treatment of schizophrenia cognitive disorders [49]
**16**	6,7-dihydroisoquinolin-8(5*H*)-one (**78**) [29]	mice immunization: affinity for (*S*)-**1** (K_d_ = 1.0 µM) [29]
		patent nicotine vaccines [30]
		patent nicotine vaccines [31]
**17**	4-chloronicotinaldehyde (**89**) [35]	-
**18**	4-chloronicotinaldehyde (**89**) [35]	-
**19**	3-methoxy-6,7-dihydroisoquinolin-8(5*H*)-one (**93**) [44]	HEK cells (α2β4: EC_50_ = 87 μM; α4β4: EC_50_ = 32 μM) [44]
		rodent brain (α2β4: K_i_ = 1.0 μM; α4β4: K_i_ = 2.6 μM) [50]
		patent treatment inflammation central nervous system [51]
		patent treatment of dry eye syndrome [52]
		patent transdermal therapeutic system [53]
**20**	2,6-dichloro-4-methylnicotinic acid (**96**) [54]	-
	2,6-dichloro-4-methylnicotinic acid (**96**) [55]	
**21**	aldehyde **101** [45]	liq. chromat. (α4β2: Δml = 0.17; α3β4: Δml = −0.04) [56]
**22**	*D*-glutamic acid (*R*)-**104** [57]	binding inhibition (α4β2: < 15%; α7: 0%) [59]
**23**	*L*-glutamic acid (*S*)-**104** [60]	-
**24**	cyclooctadiene [61]	rat brain membrane (IC_50_ = 8.0 × 10^−8^ M) [61]
	cyclooctadiene [62]	rat brain membrane (IC_50_ = 5.0 × 10^−9^ M) [62]
	cyclooctadiene [63]	rat brain membrane ((α4)_2_(β2)_3_: IC_50_ = 5 nM) [66]
	*N*,*N*-diisopropylisonicotinamide [64]	(α7: K_i_ > 100000 nM; α4β2: K_i_ =1.3 ± 0.2 nM) [67]
	4-iodonicotinaldehyde [65]	α4β2 affinity ((*+*)-**24**: K_i_ = 1.29 nM; (–)-**24**: K_i_ = 350 nM) [63]
		agonist activity for α4β2 ((*+*)-**24**: K_i_ = 3.2 ± 0.4 nM) [68]
**25**	γ-butyrolactone (**128**) [38]	rat forebrain ((+)-**25**: K_i_ = 962 nM; (–)-**25**: 1360 nM) [38]
**26**	δ-valerolactone (**129**) [38]	rat forebrain ((+)-**26**: K_i_ = 318 nM; (–)-**26**: 1040 nM) [38]
**27**	pyridine derivative **144** [71]	-
**28**	nicotinaldehyde (**153**) [72]	(α4β2*: K_i_ = 10.22 ± 1.09 nM; α7*: K_i_ = 352 ± 32 nM) [72]
**29**	6-chloronicotinaldehyde (**159**) [72]	(α4β2*: K_i_ = 43 ± 4 nM; α7*: K_i_ > 1000 nM) [72]
